# Comparing the Effect of Methylphenidate and Anodal tDCS on Inhibitory Control and Working-Memory in Children and Adolescents with Attention Deficit/Hyperactivity Disorder: A Study Protocol for a Randomized, within-Subject Trial

**DOI:** 10.3390/ijerph19084575

**Published:** 2022-04-11

**Authors:** Barbara D’Aiello, Andrea Battisti, Giulia Lazzaro, Pierpaolo Pani, Pietro De Rossi, Silvia Di Vara, Italo Pretelli, Floriana Costanzo, Stefano Vicari, Deny Menghini

**Affiliations:** 1 Child and Adolescent Neuropsychiatry Unit, Department of Neuroscience, Bambino Gesù Children’s Hospital, IRCCS, 00146 Rome, Italy; barbara.daiello@opbg.net (B.D.); andrea.battisti@opbg.net (A.B.); giulia.lazzaro@opbg.net (G.L.); pietro.derossi@opbg.net (P.D.R.); silvia.divara@opbg.net (S.D.V.); italo.pretelli@opbg.net (I.P.); floriana.costanzo@opbg.net (F.C.); stefano.vicari@opbg.net (S.V.); 2 Department of Human Science, LUMSA University, 00193 Rome, Italy; 3 Department of Physiology and Pharmacology, Sapienza University, 00185 Rome, Italy; pierpaolo.pani@uniroma1.it; 4 Department of Life Science and Public Health, Università Cattolica del Sacro Cuore, 00168 Rome, Italy; 5 Centro di Riabilitazione, Casa San Giuseppe, Opera Don Guanella, 00165 Rome, Italy

**Keywords:** MPH, drug treatments, transcranial direct current stimulation, executive functions, evidence-based medicine

## Abstract

Attention-deficit/hyperactivity disorder (ADHD) is a neurodevelopmental disorder characterized by inappropriate levels of attention, hyperactivity, and impulsivity that interfere with individual functioning. The international guidelines recommend targeting ADHD-related neurochemical brain abnormalities by intervening via drug treatment, such as methylphenidate (MPH), as first choice. Drug treatments are usually associated with a huge amount of cost for families and the healthcare system, suspension for low compliance, poor long-term efficacy, and side effects. Transcranial direct current stimulation (tDCS) has been suggested as a possible noninvasive means to safely manipulate brain activity and, in turn, improve behavior and cognition in developmental ages. Several studies have shown that tDCS has the potential to improve ADHD-related cognitive deficits, but the effect of tDCS compared with MPH has never been evaluated. The aim of the present within-subject, sham-controlled, randomized proof-of-concept study is to demonstrate the positive effect of one-session anodal tDCS analogous to the MPH drug on inhibitory control and working memory in children and adolescents with ADHD. We strongly believe that this study protocol will serve to accelerate research into low-cost, drug-free, feasible interventions for ADHD.

## 1. Introduction

Emerging during childhood, attention deficit/hyperactivity disorder (ADHD) is one of the most common lifelong brain-based disorders characterized by a mixture of inappropriate levels of inattention and/or hyperactivity/impulsivity [1]. With a prevalence of ~2–7% worldwide, it significantly interferes with or reduces the quality of academic, social, or occupational functioning. Patients with ADHD frequently suffer from psychiatric comorbid conditions such as rule-breaking behaviors, substance use disorders, and mood and anxiety disorders that become more and more of a problem during adolescence and even more so in adulthood. The clinical phenotype of ADHD is also commonly associated with a range of neurocognitive dysfunctions involving atypical responses to reward/punishment contingencies, pronounced aversion to the experience of delay, attentional fluctuation, and sluggish cognitive processing speed [2].

Several studies have attributed the symptoms of ADHD primarily to a deficit in executive functions, especially working memory (WM), response inhibition, and set shifting [3,4,5,6,7,8]. Executive dysfunctions are critically dependent on the prefrontal cortex and can result from dysregulated catecholaminergic neurotransmission in the basal ganglia-thalamocortical circuit [9,10,11,12,13,14,15]. Pharmacological interventions that modulate the dysregulated catecholaminergic neurotransmission are recommended by the international guidelines (ESCAP European Guidelines), and psychostimulants, first, methylphenidate (MPH), are indicated as first-line treatment for ADHD. More than 150 randomized placebo-controlled clinical trials promote MPH as one of the most effective treatments for alleviating behavioral and cognitive symptoms, as well as for improving life outcomes in school-aged children with ADHD. Nevertheless, about 30% of patients with ADHD do not respond well to medications, show side effects, no long-term effects [16,17], and, in adolescence, adhere to treatment poorly [18]. In addition, the critical attitude of parents to pharmacotherapy pushes them to consider other treatment options, such as cognitive behavioral therapy, which however produces modest effects [19]. Last, the cost of pharmacological interventions has a huge impact on the healthcare system.

Transcranial direct current stimulation (tDCS) has been suggested as a promising technique to scaffold the key dysfunctional brain regions associated with ADHD, with the potential to alleviate the symptoms and the related cognitive deficits [20,21,22,23,24]. By placing electrodes on the scalp, tDCS generates subthreshold polarity-dependent shifts in resting membrane potentials in underlying brain regions, inducing neuroplastic aftereffects lasting for over an hour [25,26]. When combined with a stimulus or a task, tDCS can improve synaptic transmission and empower the synaptic strength effect of the neural networks activated by concomitant activities [27]. Results documented that even one anodal-tDCS session over left DLPFC causes positive effects on inhibitory control and WM compared to placebo conditions [28,29].

Considering safety, several studies have demonstrated that tDCS induces minimal side effects, which are summarized as mild tingling and itching sensations under the electrodes, predominantly in the first few seconds of the stimulation session [21,27]. A recent systematic review [30] confirmed no serious adverse effects after 747 sessions of tDCS in patients with ADHD, supporting the safety and feasibility of this technique.

With these premises, tDCS could be promoted as a valid alternative approach to drug-based treatment that may improve cognition, as well as prompt greater adherence and reduce side effects compared to pharmacological interventions [31]. To date, only 14 studies have investigated the use of tDCS on patients with ADHD [22].

To the best of our knowledge, studies that directly compare the effect of brain-based intervention (i.e., tDCS) and treatment as usual (i.e., MPH) on executive functions—especially inhibitory control and WM—are still missing in children and adolescents with ADHD. A detailed reporting of study protocols and procedures would be useful to accelerate the reproducibility of the results and to ensure the soundness of the methods.

### Research Objectives

This is a proof-of-concept study that aims at demonstrating the effectiveness of tDCS and MPH in improving executive functions in children and adolescents with ADHD. In particular, the project aims at:

1. Investigating whether one session of anodal tDCS over left DLPFC will improve inhibitory control compared to placebo condition (sham tDCS) and to MPH;
2. Exploring whether one session of anodal tDCS over left DLPFC will enhance WM compared to placebo condition (sham tDCS) and to MPH.

## 2. Materials and Methods

### 2.1. Ethical Committee

Ethical approval for the study was granted by the local research ethics committee (process number 2185_OPBG_2020) and was registered at ClinicalTrials.gov (ID: NCT04964427) on the 13 July 2021. This study will be performed following the Declaration of Helsinki. The protocol adheres to the SPIRIT guidelines (Standard Protocol Items: Recommendations for Interventional Trials).

### 2.2. Participants

Children and adolescents will be recruited at the Child and Adolescent Neuropsychiatry Unit of the Bambino Gesù Children’s Hospital in Rome. All participants and their parents will be fully informed of the procedures and the purpose of the experiment, and the principal investigator will obtain written consent from both parents and the adolescent over the age of 12 before entering the study. Participation will be on a purely voluntary basis. Only patients for whom a clinical indication has already been given for the introduction of drug therapy with MPH and who come to the hospital for the administration of the first test dose will be recruited.

The inclusion criteria will be the following: (1) participants of both genders, diagnosed with severe ADHD (combined presentation) accordingly to the Diagnostic and Statistical Manual of Mental Disorders, Fifth Edition—DSM-5 [1]; (2) an intelligence quotient (IQ) higher or equal to 85 (IQ ≥ 85); (3) age ranging from 8 years to 13 years and 11 months included; (4) having a normal or corrected-to-normal vision; (5) having carried out at least 6 months of psychosocial and psycho-behavioral interventions; and (6) drug naïve and needing drug treatment for the severity of the ADHD symptoms.

The exclusion criteria will include: (1) the presence of neurodevelopmental disorders (i.e., autism spectrum disorders) or specific psychiatric disorders (i.e., bipolar disorders, schizophrenia spectrum disorders, or adjustment disorder) as comorbid conditions; (2) having a history of neurological or medical or genetic conditions; and (3) having a basal medical condition (i.e., heart, kidney, or liver diseases) that may exclude the possibility to administer MPH.

### 2.3. Study Design

**Figure 1:**
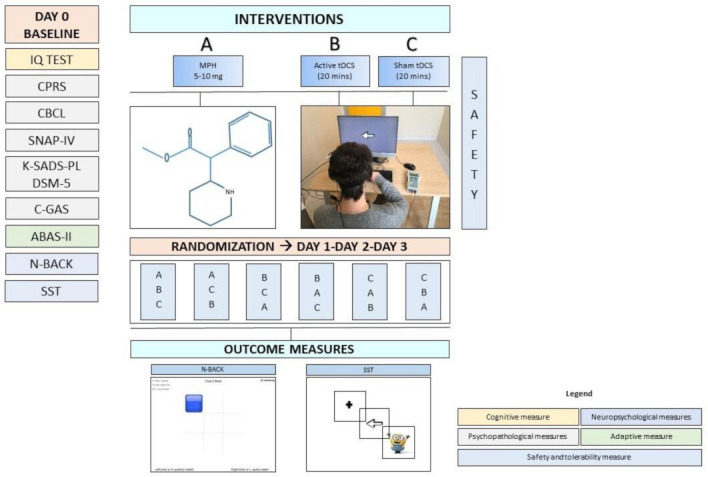
Overview of the study design. DAY 0, Baseline; DAY 1, DAY 2, DAY 3, Day of conditions administration; (**A**) single shot of active tDCS session; (**B**) single shot of sham tDCS session; (**C**) single dose of MPH (Ritalin^®^); CBCL, Child Behavior Checklist; CPRS, Conners’ Rating Scales; SNAP-IV; K-SADS-PL DSM-5, Kiddie Schedule for Affective Disorders and Schizophrenia Present and Lifetime Version for DSM-5; C-GAS, Children Global Assessment Scale (questionnaire from the K-SADS-PL DSM-5); ABAS-II, Adaptive Behavior Assessment System; N-Back; SST, Stop Signal Task; Safety and Tolerability Questionnaire.

A sham-controlled within-subjects design will be conducted. Clinical eligibility screening (Day 0) will be completed at baseline (see Figure 1). All participants will undergo an extensive neuropsychiatric evaluation in which developmental neuropsychiatrists and psychologists will investigate the cognitive and the adaptive level, the severity of ADHD symptoms, and the presence of comorbid psychiatric disorders. The following design does not involve pharmacological placebo administration as it does not aim to evaluate the effectiveness of pharmacological therapy with MPH in ADHD, which has already been established in numerous studies used for the registration of the drug on the market.

After completing baseline assessment (Day 0), participants will be exposed to three conditions with an intersession-interval of 24 h (Day 1, Day 2, Day 3, see Figure 1): (A) a single shot of active tDCS session; (B) a single shot of sham tDCS session; and (C) a single dose of MPH (Ritalin**^®^**) administered according to the National Institute for Clinical Excellence (NICE) guidelines for ADHD (NICE, 2000). The order of the conditions will be counterbalanced across participants. After recruitment, they will be assigned to one of the six possible combinations of the conditions (ABC, ACB, BAC, BCA, CBA, or CAB). We will use the stratified random sampling, based on the participants’ characteristics (e.g., age, IQ, and ADHD severity) by means of the minimal sufficient balancing method to prevent imbalances in baseline. The assignment to one of the six possible combinations will be according to a randomization order generated by a computer. The randomization information will be maintained by an independent researcher until the completion of data collection. An emergency code break envelope will be provided to the principal investigator and will only be opened in the case of an emergency, such as a serious adverse event that requires the knowledge of the interventions being taken to manage the participant’s condition.

The outcomes will be recorded at Day 0, Day 1, Day 2, and Day 3 to compare the effects of the three conditions. Specifically, at Day 0 and during the maximum peak of the tDCS effects (10 min after the start of stimulation) or MPH effects (90 min after dose administration), participants will undergo the Stop Signal Task (SST)—a measure of inhibitory control—and the N-Back task—a measure of WM. To verify that carry-over effects will not occur, the SST and the N-Back task will be performed before each session and results will be compared with those obtained at Day 0. The tDCS conditions will last approximately 40 min, including 20 min of tDCS session duration (active or sham) and 30 min of outcome measures administration, which will begin after the first 10 min of the tDCS session. The MPH condition will last approximately 2 h, including a 90 min wait time after dose administration and 30 min of outcome measures administration.

### 2.4. Interventions

#### 2.4.1. Transcranial Direct Current Stimulation

**Figure 2:**
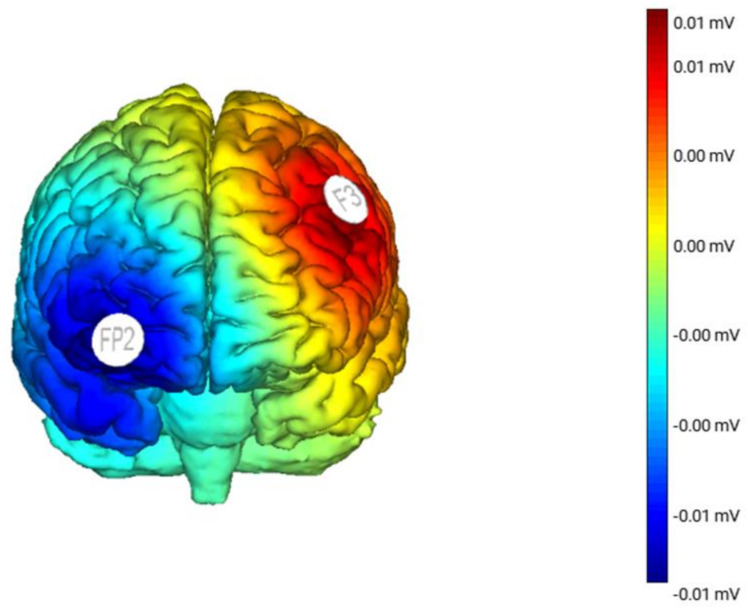
Map of electric field magnitudes in a male brain model from the frontal. The stimulating electrode will be placed over the left DLPFC, whereas the reference (cathodal electrode) will be placed above the contralateral supraorbital area with a current amplitude of 1 mA. The actual stimulation will last for 20 min, whereas the sham stimulation will consist of a current ramping up and down within 30 s.

Direct current will be delivered by a battery-driven direct current stimulator (BrainStim stimulation by E.M.S S.R.L—Bologna, Italy) via a pair of identical square (25 cm^2^) saline-soaked sponge electrodes kept firm by elastic bands. Anodal electrodes will be positioned over the left DLPFC, according to the International 10–20 System, on the sites corresponding F3, whereas the cathodal electrode will be placed above the contralateral supraorbital area (orbitofrontal cortex; OFC), corresponding to Fp2 (see Figure 2). In the active tDCS condition, the current will increase slowly during the first 30 s to 1 mA (ramp-up) and, at the end of the stimulation, the current will decrease slowly to 0 mA during the last 30 s (ramp-down). Between the ramp-up and ramp-down, constant current will be delivered for 20 min, with a density of 0.04 mA/cm^2^. In the sham tDCS condition, the stimulation will be delivered by using the same active tDCS montage, respectively left-anodal DLPFC and right-reference electrode over Fp2. Stimulation intensity will be set at 1 mA, but the current will be applied for 30 s and will be ramped down without the participants’ awareness. This placebo condition provides sensations (i.e., tingling) associated with tDCS and, therefore, it is indistinguishable by the participants from the active condition [32]. The study will be conducted in single blind: all children and their parents will be blinded to their stimulation condition.

#### 2.4.2. Methylphenidate

Depending on the age and weight of the child, a single dose of 5–10 mg of immediate-release MPH (Ritalin**^®^**) will be administered by the psychiatrist in accordance with NICE and AIFA (Agenzia Italiana del Farmaco) guidelines for the treatment of ADHD.

The indication for the prescription of the drug will be clinical and not part of an experimental study model with randomization to placebo. For this reason, the dose will not be predetermined by an algorithm involving the hospital pharmacy and the pharmaceutical company, as is the case of randomized clinical trials for drugs.

### 2.5. Clinical Eligibility Assessment

The child psychiatric examination and assessment will be conducted by experienced developmental psychiatrists and neuropsychologists. Psychiatric diagnosis will be based on developmental history, extensive clinical examination, and the semi-structured interview K-SADS-PL DSM-5 [33]. The level of severity of ADHD will be determined by clinicians according to DSM-5 criteria and classified as:

- Mild, when few symptoms beyond the required number for diagnosis are present, and symptoms result in minor impairment in social, school, or work settings;
- Moderate, when symptoms or functional impairment between “mild” and “severe” are present;
- Severe, when many symptoms are present beyond the number needed to make a diagnosis, and result in marked impairment in social, school, or work settings.

#### 2.5.1. K-SADS- PL DSM-5: The Semi-Structured Interview

Kiddie Schedule for Affective Disorders and Schizophrenia Present and Lifetime Version (K-SADS-PL) for DSM-5 (K-SADS-PL DSM-5) [33] will be submitted by trained psychiatrists and neuropsychologists. Through a comprehensive checklist of the patient’s clinical history, the clinician will ask questions of the patients and their parents separately to investigate the possible presence of current and past episodes of psychopathology, according to DSM-5 criteria [1].

Psychopathological disorders assessed by K-SADS-PL DSM-5 include the following: depressive and bipolar-related disorders; schizophrenia spectrum and other psychosis disorders; anxiety, obsessive-compulsive, and trauma-related disorders; neurodevelopmental disorders (ADHD/autism spectrum disorder); disruptive and conduct disorders; feeding and eating disorders; substance-related disorders; and elimination disorders.

#### 2.5.2. Children Global Assessment Scale

Global functioning will be assessed with the Children’s Global Assessment Scale (C-GAS) [34]. The C-GAS estimates the overall severity of disturbance (range: 0–100). Scores over 90 indicate superior functioning, whereas scores under 70 suggest impaired global functioning.

#### 2.5.3. SNAP-IV

The SNAP-IV [35] is a parent-report rating scale usually administered to evaluate comorbidity with Oppositional Defiant Disorder. It consists of 26 items that are rated on a 4-point scale (0 = no symptoms to 3 = severe symptoms). The items are divided into three subscales: inattention, hyperactivity/impulsivity, and oppositional behaviors. Subscale scores are calculated by creating an average. Higher scores represent more problem symptoms. T-scores will be used for statistical analyses.

#### 2.5.4. Child Behavior Checklist

The Child Behavior Checklist (CBCL) parent questionnaire [36] is a well-known tool for detecting behavioral and emotional problems in children and adolescents. Parents are required to evaluate the child’s behaviors and emotions during the preceding 6 months on a 3-point Likert scale for each item (0 = not true; 1 = somewhat or sometimes true; 2 = very true or often true). The hierarchic structure of the CBCL encompasses several scales, as follows: (1) syndrome scales (anxious/depressed, withdrawn/depressed, somatic complaints, social problems, thought problems, attention problems, rule-breaking behavior, and aggressive behavior); (2) broad band scales (internalizing problems, which incorporates anxious/depressed, withdrawn/depressed, somatic complaints; externalizing problems, which incorporates rule-breaking behavior, and aggressive behavior; total problems); (3) DSM-oriented scales (affective problems, anxiety problems, somatic problems, ADHD problems, oppositional defiant problems, and conduct problems); and (4) 2007 scales (sluggish cognitive tempo, obsessive-compulsive problems, and post-traumatic stress problems). The scoring software of the CBCL (Achenbach System of Empirically Based Assessment, University of Vermont: Burlington, VT, USA) generated t-scores based on the Italian standardization of the CBCL. According to the cut-off thresholds of Achenbach and Rescorla (2001), t-scores > 69 were classified as clinically relevant, t-scores between 65 and 69 were classified as borderline, and t-scores < 65 indicated non-clinical symptoms. For the internalizing problems, externalizing problems, and total problems scales, t-scores > 63 were classified as clinically relevant, t-scores between 60 and 63 were classified as borderline, and t-scores < 63 indicated non-clinical symptoms. T-scores will be used for statistical analyses.

The CBCL-Dysregulation Profile (CBCL-DP), characterized by simultaneous high values (t-scores > 70) in three syndrome scales (anxious/depressed, attention problems, and aggressive behavior), will be also calculated using the sum of t-scores of the three syndrome scales. Scores ≥ 210 are considered clinical, between 180 and 209 are in the borderline range, and ≤179 are not-clinical scores.

#### 2.5.5. Conners’ Rating Scales—Italian Adaptation

Conners’ Parent Rating Scales-Long Version Revised (CPRS) [37] are informant-report rating scales commonly used to assess behaviors related to ADHD in children. They contain 80 items that are rated on a 3-point Likert scale (0 = not true; 1 = somewhat or sometimes true; 2 = very true or often true). The t-score cut-off for relevance is >70 (very elevated). T-scores from 60 to 70 are considered high average or elevated. T-scores will be used for statistical analyses. 

#### 2.5.6. Adaptive Behavior Assessment System

Adaptive Behavior Assessment System—Second Edition (ABAS-II) [38] evaluates adaptive behavior defined as an individual’s ability to engage in skills of daily living autonomously. The ABAS-II comprises four composite scores that are made up of different domain areas: Global Adaptive Composite (GAC), Conceptual Adaptive Composite (CAC), Social Adaptive Composite (SAC), and Practical Adaptive Composite (PAC). Parents will be required to complete 232 items that are rated on a 4-point Likert scale (0 = is not able; 1 = never or rarely; 2 = sometimes when needed; and 3 = always or almost always). According to normative data, raw scores will be converted into composite scores (M ± SD: 100 ± 15). Composite scores will be used for statistical analyses.

#### 2.5.7. Non-Verbal Intelligence Quotient

The Perceptual Reasoning Index of the Wechsler Intelligence Scale for Children Fourth Edition [39] or Colored Progressive Matrices or Standard Progressive [40] will be considered as non-verbal intelligence quotient.

### 2.6. Outcome Measures

As already described, the outcome measures will be proposed to each participant individually at Day 0, before and during each condition. Specifically, to detect the maximum effects of both interventions, the outcome measures will be collected at 10 min after the start of stimulation (maximum peak for tDCS effects) [41] and 90 min after MPH dose administration (maximum peak for MPH effects as mentioned in Ritalin**^®^** label).

#### 2.6.1. Stop Signal Task 

**Figure 3:**
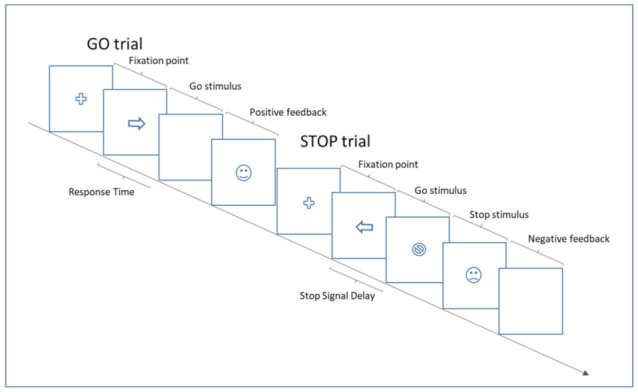
Depiction of the sequence of events in a stop-signal task.

The primary outcome of the study will be the inhibition of response (Stop Signal Reaction Time—SSRT, see Figure 3) measured with the SST [42] that consist of randomly intermixed go and stop trials (75% and 25%, respectively). The task will be performed on PsychoPy**^®^** software (Open Science Tools Ltd., Nottingham, UK), and it is structured in line with the consensus guide of SST [43]. All participants will be familiarized with the tasks before the experimental session starts. They will be performed about 10 trials of the go and no-stop task, and about 25 trials of the go no-go and the stop task. All participants will have then a clear idea of the task demand before the collection of the data starts.

All trials will begin with the presentation of a cross in the center of a computer screen. After 1500 ms, a stimulus target (go signal) will replace the cross. On go trials, children will be instructed to press the space bar as fast as possible after the go signal’s appearance. In stop trials, after a variable delay (Stop-Signal Delay, SSD), a stop signal stimulus target will appear after the go signal. Children will be instructed to refrain from responding. The SSD duration will be controlled by a simple staircase procedure (50 ms step) to keep the probability of inhibition around 50% of trials. SSD will be increased or decreased by a single step after successful or unsuccessful stopping. The stop-signal reaction time (SSRT) will be estimated (in ms) by subtracting a mean estimate of SSDs from the observed mean of the reaction times (RTs) in no-stop trials. The go no-go task will evaluate the ability to suppress a dominant response. It will consist of randomly intermixed go (75%) and no-go (25%) trials.

The output of the SST will be the following measures: SSRT, go accuracy, go RTs, SSD, and variability of reaction times (VRTs). The mean duration of the task will be 14 min.

In Figure 3, participants respond to the direction of arrows (by pressing the corresponding arrow key) in the go task. On one of the trials, the arrow is replaced by a stop symbol after a variable SSD. Positive or negative feedback follows each response.

#### 2.6.2. Visual-Spatial N-Back Task

The N-Back task is one of the most widely used culture-free tools applied to evaluate working memory. The visual-spatial condition consists of presenting a series of visual stimuli (blue boxes) in a certain location on the screen. After a training phase, participants are required to indicate whether the location of each box presented is the same as the location of the box presented *n* trials before. For example, in a 2-Back task participants have to decide whether the current location is the same as the location in trial *n* − 2. When the accuracy will be more than 80%, the difficulty of the N-Back task will increase (for example, passing from 1-Back to 2-Back). The N-Back score will be determined based on the last achieved span (where the accuracy percentage ≥80%) and the corrected percentage of the next unachieved span (where the accuracy percentage <80%). For example, when the participant achieves the 1-Back span (accuracy percentage ≥80%) and achieves only 30% accuracy in the 2-Back span, the score would be 1.3.

### 2.7. Safety Procedures

Several safety procedures will be adopted to monitor the study progress.

#### 2.7.1. Safety and Tolerability of tDCS

Symptoms and side effects will be assessed using a standard questionnaire [44] that will be completed by participants after each tDCS session. The questionnaire will list adverse effects, such as headache, neck pain, scalp pain, tingling, itching, burning sensation, skin redness, sleepiness, trouble concentrating, and acute mood change. Participants will quantify the intensity of the symptoms or side effects that will be related to tDCS (1—absent; 2—mild; 3—moderate; 4—severe).

#### 2.7.2. Safety and Tolerability of MPH

Prevention of errors and adverse effects to patients associated with MPH will be reduced to the minimum by the medical protocol for subjects under treatment with psychostimulant in line with NICE guidelines [45]. Among the most common adverse effects of MPH were reported: headache, decreased appetite, weight loss, abdominal pain, nausea and vomiting, insomnia, aggression, anxiety, depression, and hypertension. Less common are: suicidal ideation, diplopia, blurred vision, sedation and dyspnoea, and misperception. Rare, although documented, are: cardiac arrest, myocardial infarction, cerebral vasculitis, leukopenia, and thrombocytopenia. In medical emergencies, the investigator should use medical judgment and remove the subject from the immediate hazard.

#### 2.7.3. Informed Consent and Data Treatments

Before carrying out any procedure of the study, the parents or a legally authorized representative (LAR) of the subject must sign the informed consent (AIFA) and documentation of the assent (if necessary) must certify that the subject is aware of the nature of the study and the established procedures and limitations, following the International Conference on Harmonization (ICH) Good Clinical Practice (GCP) and applicable regulations.

#### 2.7.4. Protection of Risks

To minimize risks associated with tDCS, participants will be monitored throughout stimulation sessions and asked to report any discomfort. If the scalp sensation is uncomfortable, stimulation will be stopped. In the event of a headache, stimulation will be stopped. All tDCS sessions will be administered and continually supervised by a trained experimenter. tDCS side effects are minimal in children and adults, typically involving transient itching and reddening site of stimulation of the scalp on some participants [46]. However, to avoid any chance of seizure, prior history of neurological disorders is an exclusionary criterion for our study and no participants will have to have a history of seizure.

MPH. Neuropsychiatric preventive and naturalistic assessment (anamnestic history, the mental state examination, and the neurological examination) according to the AIFA guidelines for ADHD will be conducted by a developmental psychiatrist before recruitment. At that time, cardiovascular risk factors associated with MPH assumption (i.e., Brugada syndrome) will be excluded by clinicians. After this first evaluation, the participants will undergo medical examinations. Specifically, an electrocardiogram and the correction of the QT segment will be preventively evaluated by a cardiologist. Moreover, blood exams will be carried out by developmental nurses to exclude any other medical condition associated with ADHD and that may mime this disorder’s symptoms (i.e., thyroiditis). All the assessment included in this section will be at Day 0.

#### 2.7.5. Missed Sessions and Early Termination of Participation

The experimenters will register each participant’s suspension or interruption of the study. Participants will be promptly withdrawn from the study in the case of any unpredictable adverse effects. If the suspension happens during the testing session, data will be excluded from the analyses. Clinical care will not be affected.

#### 2.7.6. Study Monitoring and Data Management

The principal investigator (or the ethics committee) will identify a study monitor assigned to follow this study following this clinical trial protocol [European guidelines for Good Clinical Practice (CPMP/ICH/135/1995) and Decree-Law Italian Minister of Health, 15 July 1997]. The investigator agrees to provide reliable data and all information requested by the protocol accurately and legibly according to the instructions provided and to ensure direct access to source documents to the ethics committee representatives. If any particular circuits have to be defined, particular attention should be paid to the confidentiality of the participant’s data to be transferred.

In this case, the investigator may appoint such other individuals as he/she may deem appropriate as sub-investigators to assist in the conduct of the clinical trial following the clinical trial protocol. All sub-investigators shall be timely appointed and listed. The sub-investigators will be supervised by and under the responsibility of the investigator. The investigator will provide them with a clinical trial protocol and all necessary information.

The participant’s data will be anonymous and coded. The hard files will be placed in a closed drawer. The database will be protected by a password. The investigator will allow the monitoring with an appropriate frequency. The original documents will be available at any moment to be verified by the clinical monitor and the regulatory authority.

### 2.8. Power and Sample Size Considerations

The sample size is calculated by a priori analysis in G*Power, version 3.1.9.7 (The G*Power Team, Düsseldorf, Germany).

The estimated result will be obtained on the assumption that participants, receiving a single dose of MPH or a single session of active tDCS, will decrease the SSRT compared to the baseline, whereas participants receiving a single session of sham tDCS will not significantly change their SSRT compared to the baseline.

Because the design of this project has never been employed in children and adolescents with ADHD, we will refer to studies in which patients with ADHD completed the SST before (baseline) and after interventions (a single dose of MPH or a single session of tDCS). Regarding MPH, Rosch and collaborators’ study [47] on SSRT found a Cohen’s d effect size of 0.68 and an f effect size of 0.34. Regarding tDCS, Allenby and collaborators’ study [23] showed a Cohen’s d effect size of 0.34 and an f effect size of 0.17.

Based on these observations, we estimate an f effect size of 0.25.

With an estimated f = 0.25, α value = 0.05 (i.e., probability of false positives of 5%), and β = 0.80 (i.e., at least 80% power), the sample size is 24 as calculated using a Repeated-measures analysis of variance (RM-ANOVA) model with four within factors (baseline, active tDCS, sham tDCS, and MPH).

## 3. Data Analyses and Expected Results

The Shapiro–Wilk test will be used to test the normality of the data and Levene’s test for the homogeneity of variances. When data is normally distributed and the assumption of homogeneity will not be violated, parametric analyses will be computed. When one assumption will not be met, non-parametric tests will be conducted or a log-transformation of the distribution will be applied, if appropriate. When appropriate, sphericity will be verified by Mauchly’s sphericity test. When sphericity will not be met, Greenhouse–Geisser correction will be applied.

Categorical data will be represented as count and proportion, while continuous data as mean and standard deviation or median and range. Chi-Square analyses will be used to compare the groups on demographic and safety measures (categorical variables).

A preliminary analysis to test the effect of the four repetitions of the tasks (Day 0, Day 1, Day 2, and Day 3) for WM and SSRT will be conducted.

RM-ANOVA will be used to compare SST measures (SSRT, go accuracy, go RTs, SSD, and VRTs) and visual N-Back index, separately, with conditions (Day 0, A, B, C) as a within-subjects factor.

Post hoc comparisons will be assessed using Tukey’s honest significance test. Partial eta squares (η_p_^2^) will be used as measures of effect sizes.

We hypothesize that tDCS will improve inhibitory control and WM, as well as MPH. Specifically, we assume that a single session of anodal tDCS can induce a similar improvement from baseline as a single dose of MPH does. The sham condition should discriminate possible task learning effects.

## 4. Discussion

We have described the rationale and design of a trial conceived to compare the efficacy of a drug-based treatment (i.e., MPH) and of a brain-directed intervention (i.e., tDCS) in producing clinically meaningful impact on cognitive function in patients with ADHD.

This study will represent the first attempt to test whether a single session of anodal tDCS is as effective as a single dose of MPH, or even more so. The results will represent a significant step toward implementing large-scale multi-sessions clinical trials in the field.

The choice to compare tDCS and MPH is based on the observation that they have similar mechanisms of action at the neural level since they both modulate dopaminergic neurotransmission system in the basal ganglia-thalamocortical circuit. Specifically, MPH intervenes on dopamine active transporter DAT-1 [48,49], the primary protein responsible for clearing dopamine from the synaptic space [50], via inhibiting catecholamine reuptake inhibitor and, in turn, increasing levels of extracellular dopamine in the striatum [51,52], as well as in frontal, thalamic, and temporal brain regions [53]. Doing so, MPH would increase and stabilize catecholaminergic neurotransmission in prefrontal cortices [16], increasing activity in frontostriatal and frontoparietal networks of patients with ADHD [12]. Similarly, a recent preclinical study [54] observed a decrement of DAT-1 activity after anodal tDCS over DLPFC and MPH administration with increasing dopamine at synaptic level, especially in the hippocampus and prefrontal cortex. In line with this result, a neuroimaging study [55] in adults demonstrated that anodal tDCS over DLPFC induces extracellular dopamine release in the subcortical regions, such as the striatum and left putamen. Moreover, another neuroimaging study [56] in adults showed that one session of anodal tDCS over DLPFC increased dopamine in the right ventral striatum and that such dopamine release was significantly associated with attention enhancement. Overall, these findings suggest that anodal tDCS over DLPFC may induce a positive effect on the dopaminergic system because of the lower density of DAT-1 and may improve prefrontal-related cognitive functions usually impaired in ADHD [6].

Concerning electrode placement, the methodological decision of placing anode over DLPFC was based on the aforementioned evidence. The left lateralization of the excitatory electrode over DLPFC was supported by neuroimaging studies demonstrating the involvement of left DLPFC in response stopping [57,58], as well as in other inhibition-related phenomena [21]. The hypoactivity of these regions in ADHD is assumed to be associated with attentional, inhibitory control, and executive dysfunctions [6]. Accordingly, we selected the anodal tDCS because of its well-known excitatory potential and because of a recent meta-analysis [30] showing that anodal but not cathodal DLPFC significantly improves inhibitory control and WM in patients with ADHD. In addition, the left DLPFC (anode electrode)-right OFC (reference electrode) montage proved to be the most effective electrodes placement. In fact, the four experiments that used this montage reported better performance than the others showing that the target area of the reference electrode has an impact on the effects of tDCS on inhibitory control.

Concerning tDCS parameters, such as intensity, the selection was based on previous studies using tDCS in children with ADHD [24]. The application of 1 mA showed to be well-tolerated in children without adverse effects in previous studies [24]. Furthermore, the decision to apply 1 mA was based on the pediatric population having certain characteristics, such as smaller head size, thinner scalp, and less cerebrospinal fluid that would influence current distribution and density at the site of stimulation [59,60,61].

We choose to administer online tDCS instead of offline tDCS [62]. Indeed, it has been demonstrated that tDCS during concomitant activities enhanced synaptic strength in neural networks already activated by cognitive tasks [63]. Accordingly, a systematic review and meta-analysis [64] comparing the effects of tDCS over the DLPFC in healthy and neuropsychiatric groups showed that accuracy in online tasks was superior to offline tasks.

Concerning neuropsychological measures, we selected inhibitory control [43] and WM to have sensible and objective measure [65] of MPH [66] and tDCS [24] effect.

In the current within-subject study, the participants will be exposed in a random order to a single shot of anodal tDCS session, a single shot of sham tDCS session, and a single dose of immediate-release MPH (Ritalin*^®^*) with an interval session of 24 h, according to previous studies from our lab [67]. The potential carry-over effects have been considered. However, physiological studies demonstrated that a single tDCS session of 10–20 min results in transitory effects that last for an hour and a half at most and return to baseline after 2 h [22,68,69]. Similarly, as indicated by AIFA, even a single administration of MPH has effects, although transient. The maximum plasma concentrations of the main unesterified metabolite are reached about 2 h after administration. MPH is eliminated from plasma with an average half-life of 2 h and after oral administration, 78–97% of the dose is excreted with urine and 1–3% with feces in the form of metabolites within 48–96 h.

To ascertain the absence of carry-over effects, a preliminary analysis will be conducted by evaluating the effect of the order of the four condition repetitions.

The possibility to demonstrate the non-inferiority of tDCS to improve ADHD symptoms compared to MPH would promote its investigation as reliable and evidence-based intervention for children with ADHD. This clinical study could lay the foundation for future research perspectives on interventions for children with ADHD, speeding up the process of the understanding of this technique in pediatric rehabilitation.

Further studies are needed to compare MPH and tDCS effects in multisession double-blind treatment studies and in larger group of patients with ADHD. In addition, functional neuroimaging studies should be designed to verify that dopamine release caused by tDCS has a brain effect comparable to that of MPH.

## 5. Conclusions

We firmly believe that detailed reporting of clinical trial protocols would reduce the publication bias of future research, by prompting the reproducibility and the reliability of experimental designs.
